# Repellent Effects of Selected Organic Leaf Extracts of *Tithonia diversifolia* (Hemsl.) A. Gray and *Vernonia lasiopus* (O. Hoffman) against *Sitophilus zeamais* Motschulsky (Coleoptera: Curculionidae)

**DOI:** 10.1155/2021/2718629

**Published:** 2021-02-25

**Authors:** Stephen Maina Gitahi, Mathew Ngugi Piero, David Nganga Mburu, Alex Kingori Machocho

**Affiliations:** ^1^Department of Biochemistry, Microbiology and Biotechnology, Kenyatta University, P.O. Box 4844-00100, Nairobi, Kenya; ^2^Department of Chemistry, Kenyatta University, P.O. Box 43844-00100, Nairobi, Kenya

## Abstract

**Introduction:**

*Sitophilus zeamais* infestation is among the major setbacks to sustainable maize farming and availability. It causes an estimated annual loss of 5–10% and 20–30% of the total maize grains loss in the temperate and tropical zones, respectively. Although synthetic pesticides are quick and effective in managing crop pests, their overuse and misuse is discouraged due to their detrimental effects on human and environment. Natural pesticidal products that are extracted from plants are particularly gaining importance as an alternative to synthetic pesticides. They are available, easily biodegraded and have low toxicity to nontarget organisms. Most botanical pesticides act on insects by repelling them away from the crops in the field or in the stores. Therefore, this study aimed to determine repellency potential of organic leaf extracts of *Tithonia diversifolia* and *Vernonia lasiopus* on *S. zeamais*. *Materials and methods*. The phytochemical profile of *T. diversifolia* and *V. lasiopus* was determined using GC-MS. Laboratory-based experiments were carried out using area preference method to assess the efficacy of the extracts against weevils for a test period of 5 h. Six groups of experiments were set up with ten *S. zeamais* in each test: positive control (Actellic), negative control (solvent only), and four different experimental extract concentrations (25, 50, 75, and 100%).

**Results:**

The results indicated that *T. diversifolia* and *V. lasiopus* leaf extracts possess potent repellency effect on weevils. All the extracts simply discouraged *S. zeamais* from the treated areas recording significantly good levels of repellent activities between 26 and 96%. Furthermore, the GC-MS analysis manifested the presence of bioactive compound in the extracts which are associated with the repellency effects.

**Conclusion:**

The study scientifically confirms the traditional use of the *T. diversifolia* and *V. lasiopus* and provides important platform for further study on the extracts as bioresource of botanical repellent.

## 1. Introduction

Maize (*Zea mays*) is considered as the queen of cereals in sub-Saharan Africa (SSA). It is one of the most important crops in the world with highest production and productivity under both irrigated and rain-fed agricultural systems in the semiarid and arid tropics, especially in SSA [1]. In view of its great importance, betterment in agronomical aspects of maize should receive equally big attention globally, a lot need to be done to increase maize production and more importantly reduce loss of maize produced for food security to be realized [2]. However, there are many constraints affecting maize production.

Among the many challenges of maize production, maize is exposed to insect pest attack prior to harvest and in storage, but the storage pests form a major cause of grain loss [3]. These pests include *S. zeamais*, *S. oryzae*, *T. castaneum*, and *E. cautella*. The most common pests of stored grain are the larger grain borer and maize weevils [4]. However, *S. zeamais* is the most predominant and destructive of all these pests that need to be managed under all cost [5].

Repellents can be an effective method for control measure of weevils on stored grains. The most conventionally effective control measure of weevils is by use of synthetic repellent pesticides [6]. However, these chemicals generally tend to be expensive, with short-lived effectiveness and risky on human health among other adverse effects [7]. This critical flaws leads to ongoing research for new and effective repellents, which provide longer protection against weevils, while remaining safe, eco-friendly, and reasonably priced [8].

Medicinal plants also form an integral intervention in the management of *S. zeamais*. This is because they are generally regarded to be safe on human health and environment [7, 9]. The use of plants as storage pest repellents is very old. Some of these plants that are currently used in the management of weevils include *A. heterophyllus*, *A. sativum* and *O. basilicum*, and *Pterocarpus santalinoides* among others [10–12]. Other than their famous importance in gardens as animal feed, *T. diversifolia* and *V. lasiopus* are some of the most important plants of the Asteraceae family with numerous medicinal values. *Tithonia diversifolia* is used in the treatment of fungal infections, inflammation, pain, malaria, and diabetes among other diseases [13, 14]. *Tithonia diversifolia* is also used to control fleas, jigger, and *C. maculatus* [15–17] while *V. lasiopus* is used in management of malaria, fungal infections, worms, and ticks [18–21].

People in Embu County use these plants traditionally in the management of *S. zeamais* in stored grains. However, no scientific research on the described pesticidal activity of *T. diversifolia* and *V. lasiopus* against weevils has been published, and experimental data about their repellent properties are scanty. It is against this background that this study was conceived and designed to explore the antipyretic potential of the selected organic leaf extract of *T. diversifolia* and *V. lasiopus* against weevils.

## 2. Materials and Methods

### 2.1. Plant Sample Collection

The plants used in this study, *T. diversifolia* and *V. lasiopus*, were collected from their natural habitat in Makunguru Village, Nthawa Location, Siakago Division, Mbeere North Subcounty, Embu County, Kenya. The GPS location for *T. diversifolia* and *V. lasiopus* specimens was 0°35′39″S, 37°38′10″E and 0°35′39.51″S, 37°38′23.62″E, respectively. The fresh leaves were identified and collected from mature plants with the help of local herbalists. The folklore information obtained included the local name of the plants, part of plant harvested, season of harvesting, method of preparation, and other medicinal importance of the plants. Samples were properly sorted out, cleaned, and transported in bags to Kenyatta University, in the Biochemistry, Microbiology, and Biotechnology departmental laboratories. The plant samples were provided to an acknowledged taxonomist for botanical authentication and voucher specimens deposited at the Kenyatta University Pharmacy and Complementary/Alternative Medicine research herbaria for future reference. The specimens were assigned voucher numbers as SMG-V1/17 and SMG-V2/17 for *T. diversifolia* and *V. lasiopus*, respectively.

### 2.2. Sample Preparation and Extraction

The leaves of *T. diversifolia* and *V. lasiopus* were air dried separately under shade and room temperature for a period of two weeks. The leaves were separately ground into fine powder using a grinding electric mill and sieved using a 300 *μ*m mesh. The powder was used for organic solvent extraction following the guideline used by [22], as well as [23].

Extraction was separately done with dichloromethane and ethyl acetate 200 g of each plant leaf powders were separately soaked in 200 ml of the respective solvents for 12 hours. The extracts were decanted, and 200 ml of solvent was added and set for 24 hours. After 24 hours, filtration was done again and 200 ml of the respective solvent was added for the final extraction until 48 hours when the last filtrate was obtained. Occasional swirling was done to ensure thorough extraction. Aluminum foil and cotton wool were always used to cover the flasks to prevent escape of solvent. Muslin cloth and Whatman No. 1 papers were used for the filtrations of the extracts. The extract filtrates were then concentrated in vacuum using a Heidolph rotary evaporator, and the solvent was recovered. The concentrates were further allowed to dry to remove traces of the solvents and yield dry extracts. All the extracts were later kept in sample bottles and refrigerated at 4°C.

### 2.3. Preparation of Extract Concentrations

The plant extract concentrates were diluted with respective solvents at a concentration of 1 gml^−1^, and this was termed as stock solution (100% w/v concentration) as described by Deshmukh and Borle [24] with limited modifications. The concentrations used were as follows: 25% (w/v), 50% (w/v), 75% (w/v), and 100% (w/v). These extract concentrations were prepared as follows: the 25% (w/v) concentration was prepared by diluting 1 ml of the stock solution with 3 ml of solvent to make up 4 ml. The 50% (w/v) concentration was prepared by diluting 2 ml of stock solution with 2 ml of the solvent to make up 4 ml while for the 75% (w/v) concentration, and 1 ml of the solvent was added to 3 ml of stock solution to make up 4 ml.

### 2.4. Preparation of Maize Weevil (*Sitophilus zeamais*)

A stock culture of the maize weevil, *S. zeamais*, was initiated by collecting adult weevils from the infested maize grains and cultured in their food media (susceptible whole maize grains) under fluctuating ambient temperature and relative humidity. Two hundred unsexed adult weevils were introduced into five two-litre glass bottles with 500 g of maize. The insects were allowed to oviposit for seven days after which they were sieved out and subsequently used for the bioassay experiments. The insect stock culture was further maintained in glass bottles of two-litre capacity containing the maize grains. The weevils were reared subsequently by replacing devoured and infested grains with fresh, clean, uninfected grains in containers covered with muslin cloth to allow for air circulation and prevent escape of insects. The muslin cloths covering the containers were held in place with rubber bands. The maize dust was periodically sieved in order to prevent the growth of mould, which may lead to the caking of grains and ultimate death of the insects. *Sitophilus zeamais* breeding and the experiments were conducted at an ambient temperature of 27 ± 2°C, relative humidity of 75 ± 5.5%, and suitable photoperiod (LD 12 : 12).

### 2.5. Gas Chromatography-Mass Spectrometry (GC-MS) Analysis

Analysis of the sample was carried out using GC-MS (7890/5975 Agilent Technologies, Inc., Beijing, China) consisting of a gas chromatograph interfaced to a mass spectrometer. The GC-MS was equipped with an HP-5 MS (5% phenyl methyl siloxane) low-bleed capillary column of 30 m length, 0.25 mm diameter, and 0.25 *μ*m film thickness. For GC-MS detection, an electron ionization system with an ionization energy of 70Ev was used. The carrier gas used was helium (99.99%) at a constant flow rate of 1.25 ml/min in split mode. The injector and mass transfer line temperature were set at 250°C and 200°C, respectively, and an injection volume of 1 *μ*l was employed. The oven temperature was programmed from 35°C for 5 min, with an increase of 10°C/min to 280°C for 10.5 min, then 50°C/min to 285°C for 29.9 min with a run time of 70 min. The MS operating parameters were as follows: ionization energy, 70 eV; ion source temperature, 230°C; solvent cut time, 3.3 min; scan speed, 1666*μ*/sec; scan range, 40–550 m/*z*; and the interface temperature, 250°C. Interpretation of mass-spectrum from GC-MS analysis was performed using the central database of the National Institute Standard and Technology (NIST) which contains more than 62,000 patterns. As for the unknown components, their spectrum was compared with those which are known from the NIST library.

### 2.6. Research Design

The repellent assessment adopted a randomized controlled study design (RCD). The study used the area preference method [25]. To create the two experimental areas, Whatman No. 1 filter paper circles of 10 cm in diameter were cut into two halves. One milliliter of each extract treatment at predetermined concentrations of 25, 50, 75, and 100% was uniformly applied with a pipette to a half filter paper disc as uniformly as possible. This half filter paper circle formed the treated test area of the experiments. The other half circle was treated with solvent only to serve as negative control area. All the discs were then air dried to evaporate solvents completely. For positive control, a conventional pesticide Actellic was applied on the treated area at the recommended rate of 2 *μ*g/ml.

A full filter paper was then remade by attaching the treated halves with the untreated halves with cellotape. The treated and the untreated half-circles were hence placed contiguously on the Petri dishes, and ten weevils were carefully introduced at the center of each filter paper disc in the Petri dish and covered well. Each treatment was replicated four times. The treatments were set up into six independent treatment groups as shown in Table 1. Each of all the six experiments including the control treatments were set out with four replications.

The number of weevils in the treated (*N*_*t*_) and control (*N*_*c*_) areas of preference was counted and recorded after every one hour for five hours. These numbers were then used to calculate percent repellency (PR) of each extract by using the formula described by [26]:

(1) $$\begin{array}{c} \text{PR}\left(\text{percentage of repellency}\right)=\frac{\left(N_{c}\;–\;N_{t}\right)\;}{\left(N_{c}+\;N_{t}\right)}\times \frac{100}{1}, \end{array}$$

where *N*_*c*_ = the number of insects recorded in the control half and *N*_*t*_ = the number of insects recorded in the treated half.

The index of repellency (IR) was then calculated using the formula described by [27]:

(2) $$\begin{array}{c} \text{IR}\left(\text{index of repellency}\right)=\frac{\;2T}{T+C}, \end{array}$$

where *C* and *T* represent the number of insects on the untreated and treated areas of preference, respectively. The repellency index values were classified as repellency (values < 1), neutral (values = 1), and attractant (values > 1) [27].

### 2.7. Data Management and Statistical Analysis

The number of weevils on both experimental areas (*N*_*c*_ and *N*_*t*_) was obtained from all the different groups for each of the extracts of the two plants. The data obtained were recorded and tabulated on a broad spread sheet. Percent repellency (PR) and index of repellency (IR) were calculated using MS Excel program. The results of IR and PR were analyzed through descriptive statistics and presented as mean ± SEM. These data were subjected to inferential statistics using one-way ANOVA followed by Tukey's post hoc test for separation and pairwise comparisons of means. Unpaired Student's *t*-test was used for pairwise separation and comparison of means between different treatment groups for the two plants. The significant difference between the treatments groups were reported at *p* ≤ 0.005. All these statistical analyses were performed using Minitab version^17^ software as the statistical software. The resulting data of this study were presented in form of tables and bar graphs.

## 3. Results

### 3.1. Quantitative Phytochemical Analysis of the Selected Organic Leaf Extract of *T. diversifolia* and *V. lasiopus*

The GC-MS results of the present study showed the presence of active insect repellent compounds in *T. diversifolia* and *V. lasiopus* as indicated in Tables 2 and 3 .

### 3.2. Repellent Activity of DCM Leaf Extracts of *T. diversifolia* and *V. lasiopus* against *S. zeamais*

Generally, the DCM leaf extracts of *T. diversifolia* and *V. lasiopus* repelled *S. zeamais* with repellency index (IP) values of less than one (Tables 4 and 5 ). Overall, the average repellency activities of the *T. diversifolia* and *V. lasiopus* extracts were largely dose dependent, as they resulted in a regular pattern of repellency, from the lowest to the highest dosages. In the first hour of the experimental period, repellent activities of the *T. diversifolia* extracts were dose independent. The *T. diversifolia* extract dose of 75% evoked a greater repellency (100%) as compared to that of 100% extract concentration (95%) (Table 4).

After two hours of exposure to weevils, the *T. diversifolia* extract doses of 25 and 50% remained comparable to each other (*p* > 0.005; Table 4) but significantly different from the rest of treatment samples (*p* < 0.005; Table 4). The *T. diversifolia* extract dose of 100% caused a 100% repellent effect after the second and third hours of exposure to weevils. It was noted that these effects were not significantly different from that caused by the standard pesticide, Actellic (*p* > 0.005; Table 4). The *T. diversifolia* extract dose of 25% caused the least repellent effects on weevils at the fourth hour of the experimental period (Table 4). It was observed that the mean percentage repellent effects of the *T. diversifolia* extracts after the 5 hours experimental period ranged between 33 and 96% (Table 4). Only the *T. diversifolia* extract doses of 75% and 100% manifested repellency activities that were not significantly different from the effects caused by Actellic (*p* > 0.005; Table 4).

On the other hand, the DCM leaf extract of *V. lasiopus* remarkably repelled *S. zeamais* by an average of between 51 and 91% (Table 5) after the 5-hour duration of the experiment. The *V. lasiopus* extract concentration of 100% achieved the highest weevil repelling activity of 80% after one hour of exposure, which later decreased to 85% by the end of fifth hour of exposure to weevils (Table 5).

The least repellency activity was manifested by the *V. lasiopus* extract concentration of 50% at the fourth hour of exposure to weevils. Similar to the lower doses of the *V. lasiopus* extract (25 and 50%), the higher doses of 75% and 100% also exhibited comparable weevil repellent effects throughout the experimental period (*p* > 0.005; Table 5).

It was also observed that the *V. lasiopus* extract dose of 25% produced high repellent effects of 70% and 65% after the second and fifth hours of exposure, respectively. This effectiveness was not significantly different from the effects caused by high extract doses (75 and 100%) as well as the positive control (*p* > 0.005; Table 5).

On average, after the five hours of this experiment, it was observed that only the *V. lasiopus* extract concentration of 100% manifested repellency effects (0.09), which was not significantly different from that portrayed by synthetic pesticide, Actellic (0.02) (*p* > 0.005; Table 5).

**Figure 1:**
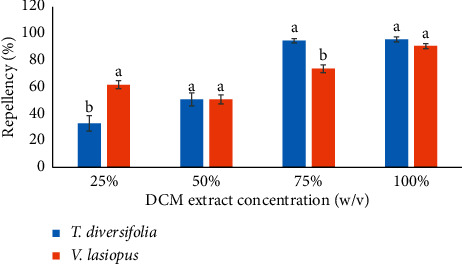
Comparison of the repellent activities (percent repellency) of the DCM leaf extracts of *T. diversifolia and (V) lasiopus* against *S. zeamais.* Bar graphs with different superscripts within the same concentration are significantly different by unpaired Student's test (*p* ≤ 0.005).

The comparison of the repellent effects of DCM leaf extracts of *T. diversifolia* and *V. lasiopus* indicated that the *V. lasiopus* extract was significantly more effective than the *T. diversifolia* extract at the extract dose of 25% (*p* < 0.005; Figure 1). However, the *T. diversifolia* extract dose of 75% manifested a significantly higher effectiveness than the *V. lasiopus* extract at similar concentration (*p* < 0.005; Figure 1). It was also evident that there was no significant statistical difference in the repellent activities of the two plant extracts at the dose level of 50 and 100% (*p* > 0.005). Both plant extracts had equal mean weevil repellency of 51% at dose of 50% (Figure 1).

### 3.3. Repellent Activity of Ethyl Acetate Leaf Extracts of *T. diversifolia* and *V. lasiopus* against *S. zeamais*

Generally, this study clearly showed that the repulsion of weevils by ethyl acetate leaf extracts of *T. diversifolia* and *V. lasiopus* advanced with increase in the extract concentration (Table 6). The repellent activities of the two plant extracts were independent of the duration of exposure to weevils. All the *T. diversifolia* extract concentrations (25%, 50%, 75%, and 100%) caused repellent activities of above 40% within the first hour of exposure to weevils (Table 6). However, at the least extract concentration level of 25%, the repellent activity gradually reduced with exposure time to 25.00% by the end of the experimental period (Table 6).

It was also evident that only the extract dose of 100% exhibited a repellent effect not statistically different from that caused by Actellic after a short time (1 hour) of exposure to weevils (*p* > 0.005; Table 6). The effectiveness of the *T. diversifolia* extract doses of 50% and 75% were comparable to each other, while the repellency caused by the dose of 25% was significantly different from the rest of treatments after such a short period of exposure to weevils (*p* ≤ 0.005; Table 6). In the second, third, and fourth hours after exposure to weevils, the *T. diversifolia* doses of 25 and 50% showed comparable effects while doses of 75 and 100% similarly showed comparable effects to each other as well as to the effects caused by the conventional pesticide, Actellic (*p* > 0.005; Table 6). All the extract concentrations showed statistically similar effects during the last hour of the test period (*p* > 0.005; Table 6).

On the other hand, the ethyl acetate leaf extract of *V. lasiopus* also showed remarkable repellent effects against maize weevils. The *V. lasiopus* extract concentration of 100% manifested the highest repellency (90%) after the second, third, and fourth hours of exposure to weevil. The lowest repellency of only 10% was manifested by the *V. lasiopus* extract concentration of 25% at the last hour of the experiment period (Table 7).

The *V. lasiopus* extract concentrations of 25% and 50% induced comparable repellent effects on *S. zeamais* throughout the test period (*p* > 0.05; Table 7). None of the *V. lasiopus* extract concentrations achieved repellent effect comparable to the effect caused by the reference pesticide, Actellic, after 1 hour of exposure to weevils (*p* ≤ 0.05; Table 7). However, the highest test concentrations of 100% demonstrated effectiveness that was comparable (*p* > 0.05; Table 7) to that of the standard pesticide throughout the rest of the experiment period. The *V. lasiopus* extract concentrations of 75% also induced repellent activity comparable to that caused by Actellic in the second and fifth hours of the test periods (*p* > 0.05; Table 7).

On average, it was observed that only the *V. lasiopus* extract dose of 100% exhibited effectiveness with repellency index value of 0.19 that was not significantly different from that portrayed by the positive control, Actellic (IP 0.02) (*p* > 0.05; Table 7).

**Figure 2:**
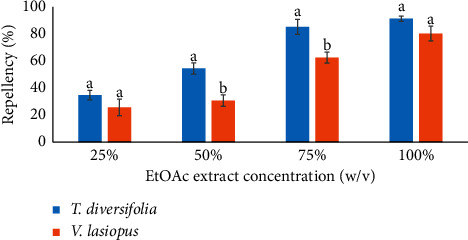
Comparison of the repellent activities (percent repellency) of the ethyl acetate leaf extracts of *T. diversifolia* and *(V) lasiopus* against *S. zeamais.* Bar graphs with different superscripts within the same concentration are significantly different by unpaired Student's test (*p* ≤ 0.005).

The comparative contrast between ethyl acetate extracts of *T. diversifolia* and *V. lasiopus* indicated that the *T. diversifolia* extract generally manifested the strongest weevil repelling potential (Figure 2). The *T. diversifolia* extract doses of 50 and 75% showed significantly more repellent abilities as compared to the *V. lasiopus* extract (*p* < 0.005; Figure 2). However, the *T. diversifolia* and *V. lasiopus* extract doses of 25 and 100% manifested statistically similar effectiveness in repellency (*p* > 0.005; Figure 2).

## 4. Discussion

This study was designed to evaluate repellent properties of crude organic extracts of *T. diversifolia* and *V. lasiopus* on *S. zeamais*. All the studied organic leaf extracts of *T. diversifolia* and *V. lasiopus* demonstrated potent repellent potential on *S. zeamais*. By the end of 5 h of the test period, it was evident that all the test samples turned out to simply discourage *S. zeamais* from attacking the grains made them craw away from the extract-treated areas. Most of the insects stayed on the untreated areas of Petri dishes and evaded the extract-treated areas. The extracts were able to induce insect repellency of between 10 and 100% within 5 hours of the experimental period.

With a minimum of 80% pesticidal action required for test substance to be considered successful [28], both of these plant organic leaf extracts largely exhibited potential repellent actions against the weevils. The repellent index value of all the organic leaf extracts of *T. diversifolia* and *V. lasiopus* against *S. zeamais* adults was lower than 1, and thus, they were classified as insect repellents and not attractants. The present finding correlated with that of other plant extracts such as *Aframomum melegueta* and *Zingiber officinale* which repelled adults *S. zeamais* [29–31].

Consistent with these findings, the hexane-ethyl acetate extracts of *C. capitatum* exhibited 90%–98% repellency activities against stored grain pests *S. oryzae, R. dominica*, and *T. castaneum.* Acetone seed extract of *Aphanamixis polystachya* showed 100% repellent effects on red flour beetles [32]. Pretheep-Kumar et al. [33] found a maximum of 91.2% of repellency with an extract of protein enriched bean flour on weevils, *S. oryzae*, after 48 h of the test period.

The high insect repellent results seen in this study are also supported by Boateng and Kusi [34] who showed that *J. curcas* seed extracts could repel up to 95% of the *C. maculatus* and *D. basalis.* Likewise, Yoon and Ahnjo [35] also reported that caraway and grapefruit successfully repelled weevils at such high rates. Acetone seed extract of *Aphanamixis polystachya* showed 100% repellent effects on red flour beetles [32]. The ethanol extracts of *Urtica dioica* and *Taraxacum officinale*, respectively, showed 99.4% and 98.8% repellency, after 48 h of the study period [36].

A number of other plants have been demonstrated to exhibit good repellent activities against *S*. *zeamais.* Ishii et al. [37] reported high susceptibility of *S*. *zeamais* to extracts and essential oils of common spices. Members of the Alliaceae family such as garlic have previously been reported to possess repellent properties on *S. zeamais* [11, 38, 39]. The extracts of *M. nodosa*, *O. surinamensis*, and *L. aurea* also showed repellent effects on *S. zeamais* [40].

The present findings correlated with repellency effects of diethyl ether extracts of *A. melegueta* and *Z. officinale* on *S. zeamais* [29]. Trivedi et al. [41] further demonstrated repellent activities of essential oils of cinnamon, clove, rosemary, bergamot, and Japanese mint against pulse beetle (*Callosobruchus chinensis*). Many other related studies have also documented repellent potential of various plants against other postharvest pests [42–44].

In contrast, Tavares and Vendramim [45] reported a lack of repellent activity of insecticidal extracts of *C. ambrosioides* on *S. zeamais*. Furthermore, contrary to the findings of the present study, [46] previously achieved very low repellent activities of herbal extracts of *T. officinale* (100%) and *U. dioica* (100%) against bean weevils. These findings do not agree with the results observed in the present work, in which all the studied extracts showed remarkable repellent activities on the target insect. This is probably due to performance of extracts derived from plants of different families from the presently studied extracts.

A free choice (area preference) bioassay model was used in this study for it is easily applicable and reliable. Similar laboratory tests were carried out using extracts of 13 plants to assess their repellent properties against the banana weevil [46]. A free choice bioassay system was also used to evaluate repellency effects of extracts and fractions from leaves of *C. capitatum* against three major stored grain insect pests, namely, *S. oryzae*, *R. dominica*, and *T. castaneum* [47].

The extract concentration ranges used in this study were within the dose ranges used by Ofuya et al. [48], Ouko et al. [11], and Acero [49, 50]. The works of Acero [49] and Acero [50] used extract concentrations of 25%, 50%, and 75% in evaluating the pesticidal properties of *A. heterophyllus* and *C. odorata* against a closely similar weevil, *S. oryzae*. The study of Ouko et al. (2017)[11] also used similar extract concentration levels in determining repellency effects of A. sativum and O. basilicum on maize weevils.

The levels of repulsion of target insects in the present study were generally proportional to the extract concentrations. An increase in extract concentration resulted in an increase in the repulsion of *S. zeamais*. This could be due to the increase in bioactive components as the concentration of the extract increased. There was no appropriate concentration of the active principle(s) at the lower extract dose levels. It is also likely that at a lower dose, there is simply not a sufficient concentration of the active principle(s).

That the effectiveness of the extracts was dependent on extract concentrations is in agreement with earlier research studies of Chaieb et al. [51], Kafle and Shih [52], Cortés-Rojas et al. [53], and Nattudurai et al. [54] among others. In a related study, Marimuthu [55] indicated plant extracts distilled from *C. citrates*, *C. zeylanicum*, *R. officinalis*, and *Z. officinale* had promising dose-dependent repellent properties against *Culex tritaeniorhynchus* and *Anopheles subpictus*. Furthermore, the nonpolar and oil leaf extracts of *Ocimum viride* also offered a dose-dependent repellent potential against *Aedes aegypti* [56]

Interestingly, the effectiveness of *V. lasiopus* extracts at low concentrations (25 and 50%) was not significantly different from the effects caused by high extract doses (75 and 100%) as well as the positive control, Actellic, during the first 2 hours of the test period. This may be due to the fact that, even at low extract concentration, the combination of repellent compounds was also in the appropriate proportional mixture to repel the target insects.

This observation was similar to the previous results for other plant extracts against different insect pests including *S. zeamais*, *T. casteneum*, and *S. oryzae* [57, 58]. Similarly, the organic extracts of *Eucalyptus globulus, Citrullus colocynthis*, and *O. basilicum* have also shown strong repellency against *S. oryzae, C. maculatus*, and *T. casteneum* at remarkably lower concentrations [59, 60].

The findings of this study demonstrated no trend with exposure time. In fact, high repellent ratings were scantly noted at both the initial and final hours of the experiment. This is could be due to a possibility that *S. zeamais* was equally sensitive to the extracts' odor even at low concentration. Other studies have also indicated such trends where repellent activities of plant extracts on insects were independent of exposure time [11]. On the contrary, Mobki et al. [58] reported that repellent activity of garlic extract to *S. zeamais* increased with duration of exposure. However, where an increase in repellency activities was noted for the first 3 hours of the test period followed by decrease in repellency is interesting. This decrease may be caused by evaporation of the active volatile compound(s).

It was evident that repellent activities were more pronounced for *T. diversifolia* than *V. lasiopus* extracts. This variation could be attributed to unequal distribution of chemical constituents within these plant species. The high repulsive activity of *T. diversifolia* extracts is an indication of higher concentration of phytochemicals with repellent activities as compared to *V. lasiopus* extracts. After all, the chemical variations in plant extracts composition are rather common even within the same species. Mainly, this depends on the type of genotype, plant organ, harvest, region, season, climatic conditions, and plant nutritional status [61, 62].

The contrasts between solvents extractives of the two plants indicated DCM extract as being significantly less potent than ethyl acetate extracts. The higher effectiveness exhibited by EtOAc leaf extracts indicated that this solvent captured more actively repellent compounds within the extract than the DCM. This finding mirrors the earlier report that EtOAc extract of *Citrullus colocynthis* and *Gnidia kaussiana* (Thymeleaceae) exhibited a higher repellent activity against cowpea weevil than hexane and methanol extracts [60].

The plant extracts acted as repellents by driving the insects away due to their smell or taste. Arthropods such as insects and mites will tend to evade areas with pungent odor [49, 50]. The repulsion of weevils by these extracts was possibly through stimulation of olfactory receptors [63]. They have several olfactory receptor cells (ORCs) in their antennae [64]. The ORCs have a coded pattern of behavior for the specific quality and quantity of semiochemicals in different complex mixtures present in their environment.

In response to an odor substance emitted into insects' environment, chemical message is decoded and integrated into the olfactory centers of the central nervous system (CNS) [65]. Hydrocarbons especially monoterpenes and oxygenated compounds such as phenols and esters determine distinctive odor of plants and hence the plants' repellent effect on insects [39, 42, 66]. To produce an odor sensation, the phytochemical substance must be volatile and its molecules must come into contact with the olfactory end organ in the insect pest [67].

This ultimately causes olfactory-induced changes in the behavior of the insects, which is of considerable importance in relation to the mechanism of insect attractancy and repellency [65]. A similar mechanism of action could have been used by the phytochemicals in *T. diversifolia* and *V. lasiopus* leaf extracts to induce repulsion activity of *S. zeamais*.

The repellent phytochemicals could also have acted by interfering with the perception of host-attractant signals. These phytochemicals could have induced excitement of receptors responsible for an opposite or competing behavior rather than food-attractancy behavior [64]. This resulted to switching of the sensory message from attraction to repulsion. Several different receptor systems were hence activated so that the normal and meaningful sensory information was “jammed” by the enhanced repellent effect and exciting the repellent (noxious substance/phytocompound) receptors [49, 64, 65].

The observed repellent activity in the present study could partly be attributed to the presence of plant volatile bioactive constituents, which are well-known repellents of insects by acting in the vapour form on the olfactory receptors [64, 68]. This has been partly due to the lack of any secure correlation between the odors of phytochemical and the chemical constitution, reactivity, physical shapes, or electrical properties of the odorous molecules [64]. Although much of explanation for repellency is generally agreed, there has been no accepted theory of the triggering process by which the odorous molecule beyond this, towards initiation and discharge of the olfactory nerve [64]. However, the mechanism of interaction of the olfactory receptors and the phytochemicals is still obscure.

The GC-MS analysis revealed a range of volatile phytochemical compounds in the tested plant extracts including alkaloids, terpenoids, fatty acids, phytosterols, and benzaldehyde, among others. These phytochemical compounds could be responsible for the observed repellency activities against the *S. zeamais*. The concentrations of the major repellant terpenoids observed in *T. diversifolia* and *V. lasiopus* organic extracts are consistent with the demonstrated properties of these plant as an insect repellent.

Terpenes are widely linked to insect repellent or attractant properties. The presence of terpenes is speculated to be associated with fragrance and repellent activities of essential oils. Several studies have also indicated terpenoids as arthropod-repellent compounds [42]. However, terpenes from ponderosa pine bark have been characterized as attractants to bark beetle, and *Ips confusus* and isothiocyanates from the seeds of crucifera are attractants to insects seeking food and site for oviposition [69].

The repellent activities of *T. diversifolia* and *V. lasiopus* extracts could have been due presence of monoterpenes. Monoterpenes such as eugenol, limonene, camphor, and thymol commonly found in basil have strong repellent activities against insects [70]. Odalo et al. [71] also found out monoterpenes components of basil (labiate) as effective repellents against *A. gambiae* (Diptera).

Widdrol is an odorous phytocompound, whose presence in the ethyl acetate leaf extract of *T. diversifolia* is likely to have contributed to the strong odor of the plant extracts. It is such smells in the extracts that are thought to drive the insects away especially because insects will always tend to evade places with such pungent odors [50]. However, it still remains difficult to make a precise association of widdrol with the repellent activities of plant extracts [72].

The presence of vanillin (a phenolic aldehyde) in the studied extracts could also have contributed to the observed repellency activities. Vanilla extract has been reported as significant insect repellent due to its distinct aroma and flavor [73]. It works well in safeguarding homes and body skin against insects such as mosquitoes, flies, and gnats [74, 75]. Vanilla extract is usually effective as an insect repellent for about half an hour. Hence, it requires reapplication more especially when exposed to very high temperatures which may cause it to evaporate quickly or in cases of being diluted with water elements such as rain [75].

The GC-MS analysis revealed that the organic leaf extracts of *T. diversifolia* and *V. lasiopus* contain odorous oleic and linoleic acids, which could also be associated with their repellent properties. Arthropods are known to release oleic and linoleic acids upon death usually referred to as “smell of death.” The smell of these compounds is believed to repel other insects, thereby keeping them away from approaching their death zone. A previous study by Ramsewak et al. [76] attributed the botanical repellent effects on *Aedes aegypti, Helicoverpa zea*, *Lymantria dispar*, *Orgyia leucostigma*, and *Malacosoma disstria* to the strong smell of oleic and linoleic acids.

The repellent activities of the extracts on adult weevils in the present study could also be linked to the presence of *α*-bulnesene in the organic leaf extracts of *T. diversifolia* and *V. lasiopus*. According to Gokulakrishnan et al. [77], *α*-bulnesene extracted from *Pogostemon cablin* exhibits repellent activities against various urban ant species. Similarly, the presence of *α*-bulnesene in *P. cablin* was associated with the plant's repellent activities against selected important vectors of mosquitoes including *Aedes aegypti*, *Anopheles stephensi*, and *Culex quinquefasciatus* [78].

The repellency of the organic leaf extracts of *T. diversifolia* and *V. lasiopus* against *S. zeamais* may be explained by the revealed presence of benzaldehyde in these extracts. Benzaldehyde is also the main chemical compound in *M. nodosa,* which was reported to be the cause of moderate repellency against *S. zeamais*, *O. surinamensis*, and *Amblyomma cajennense* [40, 79, 80].

Additionally, benzaldehyde was also extracted from *Tanaecium nocturnum* (Bignoneaceae), which caused repellency to *Sitophilus oryzae* (Coleoptera: Curculionidae), *Rhyzopertha dominica* (Coleoptera: Bostrichidae), and *T. castaneum* [81]. Furthermore, benzaldehyde has been found to be lethal on *S. zeamais* and *Tenebrio molitor* (Coleoptera: Tenebrionidae), confirming its bioactivity against stored grain pests [82].

The repellent properties of these extracts could also be attributed to specific compounds among the many that GC-MS analysis revealed in this study. However, synergistic or additive effects as a result of combination and interaction between phytochemicals cannot be ignored [83]. Furthermore, the repellent activities of the plant volatiles may not be limited only to its major constituents; it could also be due to some minor constituents or a synergistic effect of several constituents [47].

Actellic®25EC, is a broad spectrum insecticide. It is conventionally used for the control of storage pests in bulk-stored grains, bagged grains, and storage surfaces. It is also effectively used for the control of insect pests in pineapples, citrus, bananas, potatoes, and vegetables. It contains 250 g/ml pirimiphos-methyl which is taken by the insect through its respiratory system and affects the pests through its repellence effects. Although Actellic is actually a contact insecticide, it contains permethrin which was recently studied and suggested as an insecticide as well as an insect repellent [84–86]. In fact, in the US, Actellic is registered as both repellent and insecticide [84, 85]. It is worth noting that on average, after a 5 h test period, all extracts of *T. diversifolia* and *V. lasiopus* produced appreciable repellent activities against weevils, which compare well with the standard chemical Actellic. This suggests a possible mimicry of Actellic mode of action by active phytochemicals in the studied crude extracts in repelling the weevils. In conclusion, the findings of this study evidently show that the organic leaf extracts of *T. diversifolia* and *V. lasiopus* can be used as an effective repellent agent against *S. zeamais* on stored maize grains.

## Figures and Tables

**Table 1:** Treatment protocol for determination of repellency activities of *T. diversifolia* and *V. lasiopus* on *S. zeamais*.

Group	Treated area	Control area
I experimental group A	25% plant extract (w/v)	Solvent only
II experimental group B	50% plant extract (w/v)	Solvent only
III experimental group C	75% plant extract (w/v)	Solvent only
IV experimental group D	100% plant extract (w/v)	Solvent only
V positive control	Actellic	Solvent only

**Table 2:** Phytochemical analysis of insecticidal compounds in the DCM leaf extract of *T. diversifolia* and *V. lasiopus*.

Compound name	Chemical class	Molecular formula	Compound concentration (ng/g)	
			*T. diversifolia*	*V. lasiopus*
Squalene	TD	C_30_H_50_	392.50	149.90
*β*-Amyrin	TT	C_30_H_50_O	522.35	183.79
*α*-Copaene	ST	C_15_H_24_	4.32	—
Dodecanoic acid	FAD	C1_2_H_24_O_2_	7.15	—
*α*-Amyrin	TT	C_30_H_50_O	177.89	48.08
Hexadecanoic acid	FAD	C_16_H_31_O_2_	402.48	65.37
Stigmasterol	ST	C_29_H_48_O	—	13.74
Octadecanoic acid	FAD	C_18_H_30_O_2_	139.90	19.16
Indanol	P	C_9_H_10_O		5.21
Pentadecanone	FAD	C_18_H_36_O	31.94	—
Docosanoic acid	FAD	C_22_H_44_O_2_	37.88	—
p-Xylene	P	C_6_H_4_(CH_3_)_2_	4.69	—
Benzaldehyde	BD	C_7_H_8_O	8.49	—
Tetradecanal	FAD	C_14_H_28_O	9.75	5.53
Phytol, acetate	DT	C_22_H_42_O_2_	254.59	170.93
Methyl linoleate	DT	C_19_H_34_O_2_	164.40	164.40
Phytol	DT	C_20_H_40_O	159.82	051.17
Isophytol	DT	C_20_H_40_O	007.05	—
Eugenol	P	C_10_H_12_O_2_	009.53	005.30
Linalool	MT	C_10_H_18_O	007.34	—
Chondrillasterol	ST	C_29_H_48_O	—	067.52
Methyl linoleate	FAD	C_19_H_34_O_2_	164.40	043.03
Nonadecene	FAD	C_19_H_38_	—	003.18

P, phenolic; ST, sesquiterpenoid; TT, triterpenoid; DT, diterpenoid; MT, monoterpenoid; S, phytosterol, AD, aldehyde; BD, benzyl derivatives; FAD, fatty acid derivatives.

**Table 3:** Phytochemical analysis of insecticidal compounds in the ethyl acetate leaf extract of *T. diversifolia* and *V. lasiopus*.

Compound name	Chemical class	Molecular formula	Compound concentration (ng/g)	
			*T. diversifolia*	*V. lasiopus*
Nonanoic acid	FAD	C_9_H_18_O_2_	008.05	—
Squalene	TT	C_30_H_50_	448.08	122.41
*α*-Copaene	ST	C_15_H_24_	21.54	14.93
Limonene	MT	C_10_H_16_	—	005.06
Hexadecanoic acid	FAD	C_16_H_32_O_2_	789.58	321.66
Tetradecanal	FAD	C_14_H_28_O	15.46	—
Tetradecanoic acid	FAD	C_14_H_28_O_2_	45.02	13.41
*β*-Amyrin	TT	C_30_H_50_O	297.63	39.98
*α*-Amyrin	TT	C_30_H_50_O	20.78	—
Olean-12-ene acid	TT	C_30_H_50_	80.86	—
*α*-Pinene	MT	C_10_H_16_	—	004.53
Widdrol	ST	C_15_H_26_O	110.29	
Nerolidol	ST	C_15_H_26_O	86.54	56.13
Caryophyllene oxide	ST	C_15_H_24_O	19.33	11.87
*α*-Bulnesene	ST	C_15_H_24_	4.57	—
Indole	A	C_8_H_7_N	11.33	—
Indanol	P	C_9_H_10_O	16.03	—
Benzaldehyde	BD	C7H6O	5.56	8.01
Vanillin	P	C_8_H_8_O_3_	4.69	—
Naphthalene	P	C_12_H_12_	6.48	—
Methyl linoleate	FAD	C_19_H_34_O_2_	370.28	121.30
Phytol	DT	C_20_H_40_O	321.37	060.40
p-Xylene	TT	C_6_H_4_(CH_3_)_2_	—	7.42
Phytol acetate	DT	C_22_H_42_O_2_	087.95	233.70
Sabinene	MT	C_10_H_18_O	—	004.57
Eugenol	P	C_10_H_12_O_2_	016.89	—
Caryophyllene	ST	C_15_H_24_O	019.33	011.87
Linalool	MT	C_10_H_18_O	—	007.58
Citronellel	FAD	C_14_H_26_O_2_	116.29	—
Terpinen-4-ol	MT	C_10_H_18_O_0_	006.58	005.84
L-*α*-terpineol	MT	C_14_H_22_	003.47	—

P, phenolic; ST, sesquiterpenoid; A, alkaloids; TT, triterpenoid; DT, diterpenoid; MT, monoterpenoid; S, phytosterol; AD, aldehyde; BD, benzyl derivatives; FAD, fatty acid derivatives.

**Table 4:** Repellency activity of the DCM leaf extracts of *T. diversifolia* against *S. zeamais*.

Concentration (% extract)	PR (mean % ± S.E.M)^m^ with exposure time (hr.)					PR(mean ± S.E.M)^n^	IP (mean ± S.E.M)^n^
	1 hr	2 hr	3 hr	4 hr	5 hr		
25	35.00 ± 5.00^c^ (0.65)	45.00 ± 5.00^b^ (0.55)	45.00 ± 5.00^b^ (0.55)	15.00 ± 9.57^b^ (0.85)	25.00 ± 5.00^b^ (0.75)	33.00 ± 5.83^b^	0.67 ± 0.0583^a^
50	65.00 ± 5.00^b^ (0.35)	55.00 ± 5.00^b^ (0.45)	50.00 ± 5.77^b^ (0.50)	50.00 ± 5.77^b^ (0.50)	35.00 ± 5.00^b^ (0.65)	51.00 ± 4.84^b^	0.49 ± 0.0485^a^
75	100.00 ± 0.00^a^ (0.00)	95.00 ± 5.00^a^ (0.05)	95.00 ± 5.00^a^ (0.05)	90.00 ± 5.77^a^ (0.10)	95.00 ± 5.00^a^ (0.05)	95.00 ± 1.56^a^	0.05 ± 0.0158^b^
100	95.00 ± 5.00^a^ (0.05)	100.00 ± 0.0^a^ (0.00)	100.00 ± 0.00^a^ (0.00)	90.00 ± 5.77^a^ (0.10)	95.00 ± 5.00^a^ (0.05)	96.00 ± 1.87^a^	0.04 ± 0.0187^b^
Actellic	100.00 ± 0.00^a^ (0.00)	95.00 ± 5.00^a^ (0.05)	100.00 ± 0.00^a^ (0.00)	100.00 ± 0.00^a^ (0.00)	95.00 ± 5.00^a^ (0.05)	98.00 ± 1.22^a^	0.02 ± 0.0122^b^

Values followed by the same superscript within the same column are not significantly different (*p* > 0.005) determined by one-way ANOVA followed by Turkey's test. ^m^Values were means based on four extract concentrations, four replicates (*n* = 4). ^n^Values were means obtained over the 5-hour test duration (*n* = 5). Figures in parenthesis indicate the repellency index (IP < 1 for repellency, IP = 1 for neutral, and IP > 1for attractant).

**Table 5:** Repellent activity of DCM leaf extracts of *V. lasiopus* against *S. zeamais*.

Concentration (% extract)	PR (mean % ± S.E.M)^m^ with exposure time (hours) posttreatment					PR (mean ± S.E.M)^n^	IP (mean ± S.E.M)^n^
	1 hr	2 hr	3 hr	4 hr	5 hr		
25	55.00 ± 5.00^b^ (0.45)	70.00 ± 5.77^ab^ (0.30)	55.00 ± 9.57^b^ (0.45)	65.00 ± 9.57^bc^ (0.35)	65.00 ± 5.00^ab^ (0.35)	62.00 ± 3.00^bc^	0.38 ± 0.0670^ab^
50	60.00 ± 8.16^b^ (0.40)	55.00 ± 5.00^b^ (0.45)	50.00 ± 5.77^b^ (0.50)	40.00 ± 0.00^c^ (0.60)	50.00 ± 5.77^b^ (0.50)	51.00 ± 3.32^c^	0.49 ± 0.0332^a^
75	75.00 ± 5.00^ab^ (0.25)	70.00 ± 5.77^ab^ (0.30)	85.00 ± 5.0^ab^ (0.15)	70.00 ± 5.77^abc^ (0.30)	70.00 ± 5.77^ab^ (0.30)	74.00 ± 2.92^b^	0.26 ± 0.0292^b^
100	95.00 ± 5.00^a^ (0.05)	90.00 ± 5.77^a^ (0.10)	95.00 ± 5.00^a^ (0.05)	90.00 ± 5.77^ab^ (0.10)	85.00 ± 5.00^a^ (0.15)	91.00 ± 1.87^a^	0.09 ± 0.0187^c^
Actellic	100.00 ± 0.00^a^ (0.00)	95.00 ± 5.00^a^ (0.05)	100.0 ± 0.00^a^ (0.00)	100.0 ± 0.00^a^ (0.00)	95.00 ± 5.00^a^ (0.05)	98.00 ± 1.22^a^	0.02 ± 0.0122^c^

Values followed by the same superscript within the same column are not significantly different (*p* > 0.005) determined by one-way ANOVA followed by Turkey's test. ^m^Values were means based on four extract concentrations, four replicates (*n* = 4). ^n^Values were means obtained over the 5-hour test duration (*n* = 5). Figures in parenthesis indicate the repellency index (IP < 1 for repellency, IP = 1 for neutral, and IP > 1for attractant).

**Table 6:** Repellent activity of ethyl acetate leaf extracts of *T. diversifolia* against *S. zeamais*.

Concentration (% extract)	PR (mean % ± S.E.M)^m^ with exposure time (hours) posttreatment					PR (mean ± S.E.M)^n^	IP (mean ± S.E.M)^n^
	1 hr	2 hr	3 hr	4 hr	5 hr		
25	40.00 ± 0.00^c^ (0.60)	45.00 ± 9.57^b^ (0.55)	35.00 ± 5.00^b^ (0.65)	30.00 ± 5.77^b^ (0.70)	25.00 ± 5.00^b^ (0.75)	35.00 ± 3.54^b^	0.65 ± 0.0354^a^
50	65.00 ± 5.00^b^ (0.35)	50.00 ± 5.77^b^ (0.50)	50.00 ± 5.77^b^ (0.50)	45.00 ± 5.00^b^ (0.55)	65.00 ± 5.00^a^ (0.35)	55.00 ± 4.18^b^	0.45 ± 0.0418^a^
75	65.00 ± 5.00^b^ (0.25)	95.00 ± 5.00^a^ (0.05)	90.00 ± 5.77^a^ (0.10)	85.00 ± 9.57^a^ (0.15)	95.00 ± 5.00^a^ (0.05)	86.00 ± 5.57^a^	0.14 ± 0.0557^b^
100	90.00 ± 5.77^a^ (0.10)	85.00 ± 5.00^a^ (0.15)	95.00 ± 5.00^a^ (0.05)	95.00 ± 5.00^a^ (0.05)	95.00 ± 5.00^a^ (0.05)	92.00 ± 2.00^a^	0.08 ± 0.0200^b^
Actellic	100.00 ± 0.00^a^ (0.00)	95.00 ± 5.00^a^ (0.05)	100.0 ± 0.00^a^ (0.00)	100.0 ± 0.00^a^ (0.00)	95.00 ± 5.00^a^ (0.05)	98.00 ± 1.22^a^	0.02 ± 0.0122^b^

Values followed by the same superscript within the same column are not significantly different (*p* > 0.005) determined by one-way ANOVA followed by Turkey's test. ^m^Values were means based on four extract concentrations, four replicates (*n* = 4). ^n^Values were means obtained over the 5-hour test duration (*n* = 5). Figures in parenthesis indicate the repellency index (IP < 1 for repellency, IP = 1 for neutral, and IP > 1for attractant).

**Table 7:** Repellent activity of ethyl acetate leaf extracts of *V. lasiopus* against *S. zeamais*.

Concentration (% extract)	PR (mean % ± S.E.M)^m^ with exposure time (hours) posttreatment					PR (mean ± S.E.M)^n^	IP (mean ± S.E.M)^n^
	1 hr	2 hr	3 hr	4 hr	5 hr		
25	45.00 ± 5.00^bc^ (0.55)	30.00 ± 5.77^c^ (0.70)	30.00 ± 5.77^b^ (0.70)	15.00 ± 5.00^c^ (0.85)	10.00 ± 5.77^c^ (0.9)	26.00 ± 6.20^c^	0.74 ± 0.0620^a^
50	30.00 ± 5.77^c^ (0.7)	45.00 ± 5.00^bc^ (0.55)	25.00 ± 5.00^b^ (0.75)	20.00 ± 8.16^c^ (0.80)	35.00 ± 5.00^bc^ (0.65)	31.00 ± 4.30^c^	0.69 ± 0.0430^a^
75	65.00 ± 5.00^b^ (0.35)	65.00 ± 5.00^ab^ (0.35)	50.00 ± 5.77^b^ (0.50)	60.00 ± 0.00^b^ (0.40)	75.00 ± 5.00^a^ (0.25)	63.00 ± 4.06^b^	0.37 ± 0.0406^b^
100	70.00 ± 5.77^b^ (0.30)	90.00 ± 5.77^a^ (0.10)	90.00 ± 5.77^a^ (0.10)	90.00 ± 5.77^ab^ (0.10)	65.00 ± 5.00^ab^ (0.35)	81.00 ± 5.57^ab^	0.19 ± 0.0557^bc^
Actellic	100.00 ± 0.00^a^ (0.00)	95.00 ± 5.00^a^ (0.05)	100.0 ± 0.00^a^ (0.00)	100.0 ± 0.00^a^ (0.00)	95.00 ± 5.00^a^ (0.05)	98.00 ± 1.22^a^	0.02 ± 0.0122^c^

Values followed by the same superscript within the same column are not significantly different (*p* > 0.005) determined by one-way ANOVA followed by Turkey's test. ^m^Values were means based on four extract concentrations, four replicates (*n* = 4). ^n^Values were means obtained over the 5 hour test duration (*n* = 5). Figures in parenthesis indicate the repellency index (IP < 1 for repellency, IP = 1 for neutral, and IP > 1for attractant).

## Data Availability

No data were used to support this study.
